# Adequate Dietary Diversity Versus Suboptimal Diet Quality: The Paradox of Food Insecurity Among International Students in Hungary

**DOI:** 10.3390/nu18060946

**Published:** 2026-03-17

**Authors:** Zibuyile Mposula, Tünde Pacza, Judit Szepesi, Morris Mbuthia Wagaki, Endre Máthé

**Affiliations:** 1 Doctoral School of Nutrition and Food Science, Faculty of Agricultural and Food Sciences and Environmental Management, University of Debrecen, Böszörményi Str. 128, 4032 Debrecen, Hungary; 2 Institute of Nutrition Science, Faculty of Agricultural and Food Sciences and Environmental Management, University of Debrecen, Böszörményi Str. 128, 4032 Debrecen, Hungarymorris.wagaki@outlook.com (M.M.W.); 3 Department of Life Sciences, Faculty of Medicine, Vasile Goldis Western University of Arad, L. Rebreanu Str. 86, 310414 Arad, Romania

**Keywords:** food insecurity, dietary diversity, international students, food variety, Hungary, university health, nutrition equity

## Abstract

**Background/Objectives:** Food insecurity remains a growing public health concern among university populations, particularly international students who often face financial constraints, limited social support, and cultural adaptation challenges. This study investigated the association between food insecurity and dietary diversity among international students in Hungary, a population for whom empirical evidence remains limited. **Methods:** A cross-sectional survey was conducted among 380 international university students using a structured questionnaire comprising sociodemographic items, the Food Insecurity Experience Scale (FIES), and a quantitative Food Frequency Questionnaire (FFQ). Dietary diversity was assessed through Food Group Diversity Score (FGDS) and Food Variety Score (FVS). Statistical analyses included chi-square tests, ANOVA, correlation analyses, and multiple regression using IBM SPSS 28.0. **Results:** Overall, 62% of participants experienced food insecurity, with 25% moderately and 20% severely food insecure, while 17% were classified as mildly food insecure. While 97% achieved high dietary diversity, only 31% exhibited high food variety. Group comparisons indicated differences in FGDS across food security categories (*p* = 0.006), whereas FVS did not differ significantly (*p* = 0.411). In multivariable regression models adjusting for socioeconomic and behavioural factors, food security status was not independently associated with FGDS or FVS. However, scholarship status, monthly income, employment, and meal skipping were significant predictors of dietary diversity indicators. **Conclusions:** These findings suggest that while food insecurity is prevalent among international students, socioeconomic resources and behavioural factors may play a more prominent role in shaping dietary diversity outcomes. Universities and policymakers should prioritise equitable food access, culturally inclusive meal services, and ongoing monitoring of student food security to promote nutrition equity and academic well-being.

## 1. Introduction

Food insecurity is a major global public health concern that disproportionately affects vulnerable populations, including university students [1]. International students constitute a particularly at-risk subgroup due to financial constraints, abrupt dietary transitions, cultural and language barriers, limited social support networks, and precarious residency conditions in host countries [2].

Food security, as defined by the Food and Agriculture Organization (FAO), exists when individuals have consistent physical and economic access to sufficient, safe, and nutritious food to meet their dietary needs for an active and healthy life [3]. Although food insecurity has traditionally been examined in low-income households, it is increasingly recognized within higher education settings, where students often experience financial strain and unstable access to adequate food [4].

Global educational mobility has expanded substantially, with the number of international students increasing by approximately 176% between 2002 and 2022, reaching 6.9 million worldwide [5,6]. This growth has introduced new dimensions to food and nutrition security research, as international students frequently encounter disrupted food access and altered consumption patterns in host countries [7]. Studies across North America, Europe and Asia report food insecurity prevalence rates among university students ranging from 20% to over 50%, depending on socioeconomic and contextual factors [8,9,10]. These findings highlight university students as a potentially overlooked vulnerable group in nutrition research [11].

Dietary diversity, defined as the number of distinct food groups consumed over a specified reference period, is widely used as an indicator of dietary quality and nutritional adequacy [12,13]. Measures such as the Dietary Diversity Score (DDS) capture the breadth of food consumption and adherence to dietary guidelines [13]. Similarly, food variety, reflecting the number of different food items consumed within and across food groups, has been associated with improved micronutrient intake and overall dietary adequacy [14,15]. These indicators are particularly useful for assessing diet quality in populations with constrained resources.

Food insecurity is consistently associated with reduced dietary diversity; monotonous food choices; and lower consumption of fruits, vegetables, and animal-source foods, alongside greater reliance on inexpensive, energy-dense and ultra-processed products [16]. Such dietary compromises contribute to inadequate micronutrient intake and may exacerbate the double burden of malnutrition, increasing risks of both undernutrition and overweight/obesity [17]. Among university students, these patterns may be intensified by limited cooking facilities, inadequate food storage, time constraints, and financial pressures [11].

Among international students, food insecurity often manifests as limited access to nutritious and culturally familiar foods, greater reliance on inexpensive or ultra-processed products, and reduced dietary diversity and variety. These dietary compromises have been linked to adverse health and academic outcomes, including compromised nutritional status, psychological distress, reduced cognitive function, and poorer academic performance [18]. Adapting to a new food environment may also involve navigating differences in pricing, availability, cultural preferences, and unfamiliar food labelling or retail systems, which can substantially affect dietary choices and overall diet quality [19].

International students frequently face challenges related to unfamiliar food systems, limited access to preferred foods, and social isolation [20]. Combined with financial pressures and limited social support, these factors may lead to irregular meal patterns, skipped meals, nutrient deficiencies, and compromised diet quality [21]. In Hungary, where international enrolment continues to rise [22], such challenges are further influenced by differences in food environments and financial strain. The Hungarian food system, characterized by seasonal availability, pricing structures, and distinct retail formats, may present adaptation difficulties for students unfamiliar with Central European markets [20]. In the 2020/2021 academic year, 40,000 international students were enrolled in Hungarian higher education, accounting for 14% of the total student population [23].

Recent studies in Central Europe have identified food insecurity as a strong predictor of psychological distress among international students in Hungary [24], while research in Poland reported prevalent poor dietary and lifestyle behaviours among foreign students [25]. However, empirical evidence examining the relationship between food insecurity and dietary diversity in Central and Eastern European higher education remains limited. Most studies focus on psychosocial, economic, or mental health outcomes, with less emphasis on comprehensive dietary quality indicators [24,25]. This gap restricts understanding of how food insecurity influences diet quality among multicultural and mobile student populations. Linking food security to dietary diversity allows for the assessment of both diet quantity and quality, thereby informing targeted interventions.

Therefore, this study investigated the association between food insecurity and dietary diversity among international students in Hungary. The primary aim was to evaluate the association between food security status and dietary diversity indicators, specifically the FGDS and FVS. Secondary aims were to examine whether these associations differed according to sociodemographic and behavioural characteristics. Given the cross-sectional design, analyses focused on identifying associations rather than causal relationships.

## 2. Materials and Methods

### 2.1. Study Design and Participants

This cross-sectional study was conducted among international students enrolled in various higher education institutions in Hungary. Participants were enrolled in 11 Hungarian higher education institutions, predominantly large public universities. The largest proportion was from the University of Debrecen (38.7%), followed by Corvinus University of Budapest (13.7%) and the Hungarian University of Agriculture and Life Sciences (10.5%), with smaller representation from other institutions. Participants were eligible if they were international students enrolled at a Hungarian higher education institution, aged between 18 and 40 years, currently studying in Hungary, and able to understand and complete the questionnaires in English. A combination of purposive and convenience sampling approaches was used. Participants were recruited through university mailing lists, student associations, and on-campus data collection events. Purposive sampling was used to recruit international students who met predefined eligibility criteria, including enrolment at Hungarian higher education institutions and exposure to the Hungarian food environment, while aiming to capture diversity across cultural backgrounds, scholarship status, and socioeconomic conditions relevant to the study objectives. Data were collected between June 2022 and January 2024 using electronic and face-to-face surveys. Participation was voluntary and anonymous, and informed consent was obtained prior to questionnaire completion.

Although validated instruments were used in this study, the full survey package was pilot tested among 10 international students who met the eligibility criteria but were excluded from the main study sample. The pilot testing was conducted to assess clarity, cultural relevance, and comprehensiveness of the questionnaires within the Hungarian higher education context. Minor wording adjustments were made to improve clarity where necessary, and no substantial modifications to the validated instruments were introduced.

### 2.2. Ethical Considerations 

This study was approved by the Research Ethics Committee of the Institute of Nutrition and Food Science at the University of Debrecen. Written informed consent was obtained prior to data collection. Confidentiality, anonymity, and participants’ right to withdraw without consequence were assured.

### 2.3. Instruments and Measures

#### 2.3.1. Sociodemographic Questionnaire 

A structured sociodemographic questionnaire collected data on the participants’ gender, age, continent of origin, accommodation status, employment, scholarship status, level of study, and estimated monthly income. The tool was adapted from previous studies and adjusted for cultural and regional relevance [26].

#### 2.3.2. Anthropometric Measurements

Anthropometric measurements were obtained using calibrated instruments following standardised procedures. Body weight was measured to the nearest 0.1 kg using a digital fitness scale (Vivamax Multicare Fitness Scale, model GYVBF2, Vivamax Health Products Ltd., Dunakeszi, Hungary). Participants were measured wearing light clothing and without shoes. Height was measured to the nearest 0.1 cm using a portable mechanical stadiometer (Seca Bodymeter 206, Seca GmbH & Co. KG, Hamburg, Germany), with participants standing upright, barefoot, and with the head positioned in the Frankfort horizontal plane.

Body mass index (BMI) was calculated as body weight in kilograms divided by height in metres squared (kg/m^2^). Waist circumference and hip circumference were measured using a standard non-elastic measuring tape following established anthropometric procedures. Waist circumference was measured at the midpoint between the lowest rib and the iliac crest, while hip circumference was measured at the widest portion of the buttocks. Waist-to-hip ratio (WHR) was calculated by dividing waist circumference by hip circumference.

Blood pressure was measured using an automated upper-arm digital blood pressure monitor (Beurer BM 26, Beurer GmbH, Ulm, Germany). Measurements were taken twice, with participants seated and resting for several minutes prior to assessment.

#### 2.3.3. Food Frequency Questionnaire (FFQ) 

Dietary diversity was assessed using a quantitative FFQ comprising 111 individual food items grouped into 12 major food categories [27]. The FFQ was adapted from previous studies [28] and adjusted to reflect the Hungarian food environment and culturally diverse dietary practices of international students. Prior to implementation, the instrument was pre-tested among a subset of international students to ensure clarity, cultural relevance, and comprehensiveness.

Participants were asked whether they had consumed each food item during the previous seven days, with responses recorded as “yes” or “no.” The responses were used to compute two key indicators:

- FGDS: Calculated by summing the number of food groups consumed over seven days across 12 groups (cereals and grains; roots and tubers; legumes; dairy products; meat and poultry; fish and seafood; eggs; fruits; vegetables; fats and oils; sweets and snacks; sugar-sweetened beverages) [29].
- FVS: Total number of individual food items consumed, reflecting diversity within and across food groups [30].

Both scores were treated as continuous variables and categorised into low, medium, and high levels for comparative analyses.

Definitions:

- Food group diversity categories: Classification of individuals based on the number of distinct food groups consumed over seven days.
- Low/Medium/High dietary diversity (LDD/MDD/HDD): Consumption of 0–3, 4–6, and ≥7 food groups, respectively, over seven days [31].
- Low/Medium/High food variety (LFV/MFV/HFV): Consumption of 0–33, 34–67, and 68–101 distinct food items, respectively, over seven days.

#### 2.3.4. Food Insecurity Experience Scale (FIES)

Food insecurity was measured using an adapted version of the FIES [32], assessing students’ experiences during the past 30 days. Items represented progressive severity, from anxiety about food access to skipping meals or going a whole day without food.

Participants responded using a four-point frequency scale:

- Never (1);
- Rarely (2);
- Sometimes (3);
- Often (4).

Based on FAO thresholds, participants were initially classified into four categories: food secure (0), mildly food insecure (1–3), moderately food insecure (4–6), and severely food insecure (7–8). For analysis and reporting, respondents classified as food secure and mildly food insecure were combined into a single category (food secure), consistent with FAO reporting practices. Participants were therefore classified as food secure, moderately food insecure, or severely food insecure [33]. Internal reliability was satisfactory (Cronbach’s α = 0.90), indicating good consistency.

### 2.4. Data Analysis 

Data were entered and screened in Microsoft Excel^®^ and analysed using IBM SPSS Statistics (version 28.0; IBM Corp., Armonk, NY, USA). Statistical significance was set at *p* < 0.05 (two-tailed).

Descriptive statistics, including means, standard deviations, medians, interquartile ranges, and percentages, were used to summarise sociodemographic characteristics, dietary diversity indicators, and food security status. Internal consistency of the FIES was assessed using Cronbach’s α (Table S1; Figure S1). Pearson correlation analyses were conducted to examine associations between FIES scores, FGDS, FVS, monthly income, and food expenditure.

Group differences were assessed using chi-square tests for categorical variables and one-way analysis of variance (ANOVA) for continuous variables, with Tukey post hoc tests applied where appropriate. Effect sizes were reported using Cramer’s V for chi-square tests and η^2^ for ANOVA. Food security status (FSS) was analysed as a categorical variable with three levels: food secure, moderately food insecure, and severely food insecure.

Multiple linear regression analyses were conducted to examine associations between FSS and dietary diversity indicators (FGDS and FVS). Independent variables were entered simultaneously using the Enter method. Standardised regression coefficients (β), *p*-values, and model fit indices (R^2^ and adjusted R^2^) were reported.

Age was categorised into three groups (18–25, 26–30, and >30 years), with the 18–25-year group used as the reference category. Monthly income and monthly food expenditure were entered as continuous variables (in euros) to preserve variability. Funding status was classified as scholarship-funded or non-scholarship funded. Employment status was categorised as full-time employment, part-time employment, self-employment, or unemployed, with unemployed students used as the reference category.

Model assumptions for regression analyses, including normality, linearity, homoscedasticity, and multicollinearity, were evaluated using residual diagnostics and variance inflation factors (VIF < 5), indicating no evidence of problematic multicollinearity.

## 3. Results

### 3.1. Participant Characteristics

In total, 380 international students were included in this study. The gender distribution was balanced (51% male, 49% female), and most were aged 18–25 years (55%). The majority were African/African American (58%) or Asian (26%), with most residing in the Northern Great Plain (38%) or Central Hungary (36%). Accommodation arrangements varied, with most students living in paid shared housing (45%), followed by renting alone (26%) and university dormitories (24%), while only a small proportion reported living in shared housing free of charge (5%). More than half were scholarship recipients (55%), with relatively equal representation of undergraduate (53%) and postgraduate (47%) students. Employment levels were low, with 71% unemployed, 23% working part-time, 5% employed full-time, and 2% self-employed. Most reported monthly incomes between €425–€530 (30%) or €290–€400 (24%), and only 7% reported earning more than €795 (Table 1). Sociodemographic and socioeconomic characteristics are presented in Table 1, while food security and dietary diversity indicators are summarised in Table 2. The distribution of dietary diversity indicators are presented in Table 3, whiles food access and dietary practices are presented separately in Table 4 to improve clarity and thematic organisation.

### 3.2. Prevalence of Food Insecurity and Dietary Diversity Among Participants

As shown in Table 2, 38% of participants were classified as food secure, 31% as moderately food insecure, and 31% as severely food insecure. Regarding dietary diversity, 97% of participants were classified as having high dietary diversity, while 3% had medium dietary diversity. For food variety, 31% of participants were classified as having high food variety, 50% medium food variety, and 19% low food variety, respectively.

### 3.3. Distribution of Dietary Diversity Scores Among Participants

To describe the distribution of dietary diversity indicators within the cohort, FGDS and FVS were examined. As presented in Table 3, both variables were non-normally distributed (Shapiro–Wilk *p* < 0.001); therefore, medians and interquartile ranges (IQR) are reported. Median (IQR) values were 11.0 (10.0–12.0) for FGDS and 53.0 (37.0–78.8) for FVS.

Subgroup comparisons of FGDS and FVS across student characteristics are presented in Supplementary Table S3. Higher FGDS and FVS values were generally observed among scholarship recipients, students with higher monthly income, and those who were employed. Differences were also observed according to level of study, accommodation type, and Hungarian region.

**Table 3:** Distribution of dietary diversity indicators among international students in Hungary (*n* = 380).

Variable	Median (IQR)	25th Percentile (Q1)	50th Percentile (Median)	75th Percentile (Q3)	Min.	Max.
FGDS	11.0 (10.0–12.0)	10.0	11.0	12.0	9	12
FVS	53.0 (37.0–78.8)	37.0	53.0	78.8	25	97

IQR = interquartile range (Q3 − Q1). All variables were non-normally distributed (Shapiro–Wilk *p* < 0.001); therefore, medians and IQRs are reported. Higher FGDS and FVS indicate greater food group diversity and food variety, respectively.

### 3.4. Food Access and Dietary Practices Among Participants

The majority of participants reported a monthly food expenditure of €50–€105 (51%) and identified mainstream supermarkets as their primary food source (55%), followed by discount supermarkets (37%). Grocery shopping most commonly occurred 4–5 times per month (51%), and 62% reported consistently preparing their own meals. Meal skipping was reported by 60% of participants (Table 4).

**Table 4:** Food access and dietary practices among international students in Hungary (*n* = 380).

Variable	Category	*n*	%
Monthly food expenditure (€)	€50–€105	193	51
	€130–€210	175	46
	≥€235	12	3
Primary food source	Discount supermarkets	142	37
	Mainstream supermarkets	208	55
	Local/ethnic shops	22	6
	Online/other sources	8	2
Shopping frequency (groceries/month)	1 time	25	7
	2–3 times	120	32
	4–5 times	192	51
	>5 times	43	11
Eating out frequency (per month)	0	8	2
	1–2	123	32
	3–4	143	38
	≥5	103	27
Food preparation	Always prepare own food	237	62
	Do not always prepare own food	143	38
Skipping meals	Yes	229	60
	No	151	40

Percentages may not total 100% due to rounding or multiple response options.

### 3.5. Association Between Food Security Status and Sociodemographic and Behavioural Characteristics

As presented in Table 5, food security status was significantly associated with scholarship status (*p* = 0.047) and meal skipping (*p* < 0.001), with scholarship recipients more frequently classified as food secure and students reporting meal skipping more likely to experience moderate or severe food insecurity. The association between food security status and meal skipping demonstrated a large effect size (Cramer’s V = 0.91).

### 3.6. Differences in Dietary Diversity Scores Across Food Security Levels

One-way ANOVA results (Table 6) showed significant differences in FGDS across food security levels (F(2,377) = 5.18, *p* = 0.006). Mean FGDS was 11.2 ± 1.1 among food-secure participants, 10.7 ± 1.1 among moderately food insecure participants, and 10.9 ± 1.2 among severely food insecure participants. Food-secure students therefore demonstrated slightly higher FGDS compared with moderately and severely food insecure groups. No significant differences were observed in FVS across food security levels (F(2,377) = 0.89, *p* = 0.411).

### 3.7. Association Between Food Insecurity and Dietary Diversity After Adjusting for Confounders

Multiple linear regression analysis was conducted to examine associations between food security status and dietary diversity indicators after adjusting for sociodemographic, behavioural, and economic variables (Table 7). The overall regression models were statistically significant for both outcomes (FGDS: F(8,371) = 5.82, *p* < 0.001; FVS: F(8,371) = 7.10, *p* < 0.001). Food security status was not significantly associated with FGDS or FVS after adjustment. Higher dietary diversity scores were observed among students receiving scholarships and those with higher monthly incomes**, while employment status was positively associated with FVS.** Students who reported skipping meals were significantly associated with lower dietary diversity scores. Full regression coefficients, 95% confidence intervals, and collinearity diagnostics are presented in Supplementary Tables S4 and S5.

## 4. Discussion

This study examined how food insecurity intersects with socioeconomic resources, behavioural adaptations, and dietary patterns to shape dietary diversity among international students in Hungary. Using prevalence estimates and multivariable regression analyses, this study examined the relationship between food insecurity, socioeconomic characteristics, and dietary diversity indicators among international students. More than 60% of participants experienced some degree of food insecurity, yet most maintained high food group diversity, suggesting that apparent dietary adequacy may mask underlying constraints in food variety and dietary quality. Although food insecurity showed associations with dietary indicators in descriptive and bivariate analyses, it was not significantly associated with food group diversity or food variety after adjusting for socioeconomic and behavioural factors. Across analyses, socioeconomic resources such as scholarship support, income, and employment status emerged as more consistent predictors of dietary diversity, while behavioural factors such as meal skipping were negatively associated with dietary outcomes. Collectively, these findings reveal a complex and layered set of economic, behavioural, and structural influences on students’ dietary behaviours, highlighting that nutritional vulnerability can persist even within highly educated and mobile populations, consistent with emerging evidence from other European and global contexts [34].

### 4.1. Food Insecurity and Its Determinants

The prevalence of food insecurity (62%) observed in this study is considerably higher than reports among university students in many high-income settings. For instance, studies from the United States, Australia, and Canada have found prevalence rates ranging between 35–48% [35,36]. Comparable evidence from European contexts remains limited, but research among international or migrant students similarly points to heightened vulnerability due to financial instability, limited access to social benefits, and cultural or linguistic barriers [37,38].

The significant association between scholarship status and food security found here highlights the protective role of institutional financial aid. Scholarship recipients were more likely to be food secure, aligning with findings from previous studies which noted that structured financial support programmes reduce the odds of food insecurity among students [39,40]. Moreover, meal skipping emerged as a strong behavioural correlate of food insecurity. Students facing limited resources frequently adopted coping mechanisms such as meal skipping or portion reduction. Similar patterns have been documented among low-income youth in both developed and developing regions [41,42]. Together, these findings position food insecurity among international students not merely as a financial issue, but as a structurally mediated condition shaped by institutional support, access to resources, and behavioural coping strategies.

### 4.2. Dietary Diversity and Variety Patterns

While most participants exhibited high food group diversity, the moderate variety within groups suggests dietary monotony, possibly reflecting constrained budgets and limited food access. This duality between high dietary diversity but low food variety has also been noted among student and migrant populations, where individuals manage to cover major food groups but repeatedly consume inexpensive staples within each group [43,44]. In the present study, differences in food group diversity across food security categories were observed in group comparisons, although food security status was not independently associated with dietary diversity indicators after adjustment for socioeconomic and behavioural variables in regression models. This pattern suggests that structural and economic factors may partly mediate the relationship between food insecurity and dietary diversity among international students. Similar findings have been reported in studies indicating that socioeconomic resources, rather than food insecurity status alone, often shape dietary quality and food access among student populations [45,46]. By jointly examining food group diversity and within-group food variety, this study extends prior work by illustrating how conventional diversity indicators may obscure subtle but nutritionally meaningful forms of dietary restriction. Standard dietary diversity measures are sensitive to food group classification structure, and broadly defined food groups may overestimate adequacy when within-group consumption is limited [31]. The concurrent use of FGDS and FVS in the present study was intended to address this methodological consideration by differentiating between food group presence and within-group variety. Such dietary patterns may have important implications for long-term health outcomes. Lower intake of fruits, vegetables, and protein-rich foods can lead to a deficiency in vital micronutrients like iron, calcium, vitamin D, and B-complex vitamins. In the long run, consistently monotonous diets with a heavy reliance on cheap staple foods might weaken immune function, cause fatigue, decrease cognitive abilities, and heighten the risk of developing diet-related non-communicable diseases [47].

### 4.3. Socioeconomic and Behavioural Predictors of Dietary Diversity

Consistent with prior literature, socioeconomic variables such as scholarship status, monthly income, and employment strongly influenced dietary outcomes. Students with part-time jobs reported significantly higher food variety, supporting the view that stable income streams facilitate access to a wider range of food options [48]. The positive association between scholarship status and both FGDS and FVS indicates that financial aid may buffer food insecurity by reducing economic stress and expanding dietary options.

Behavioural factors also contributed meaningfully to dietary outcomes. Meal skipping was inversely associated with both food group diversity and food variety, suggesting that irregular eating patterns may reflect both coping responses to financial constraints and broader disruptions to regular dietary intake. Similar findings have been reported among university students experiencing financial strain, where meal skipping is frequently used as a strategy to manage limited food budgets [49]. Importantly, the persistence of socioeconomic predictors such as income, employment, and scholarship status in the adjusted regression models indicates that financial and structural resources play a central role in shaping dietary diversity among international students. While food insecurity remains an important contextual indicator of vulnerability, these findings suggest that economic capacity and institutional support mechanisms may ultimately determine the range and diversity of foods available to students [50].

Overall, this study advances understanding of food insecurity among international university students by demonstrating that constrained dietary diversity can persist despite apparent food group adequacy and moderate income levels. By integrating behavioural, socioeconomic, and dietary pattern analyses, the findings indicate that food insecurity is embedded within broader structural and economic conditions affecting students’ access to food. These results emphasise the need for campus-based interventions that move beyond emergency food provision to address affordability, dietary quality, and structural barriers to food access, particularly for culturally diverse student populations studying abroad.

## 5. Public Health and Policy Implications

The findings of this study have important implications for university-level and national public health strategies. The high prevalence of food insecurity among international students, despite generally adequate food group diversity, underscores the presence of hidden nutritional vulnerability that may not be captured by conventional dietary monitoring indicators. These findings highlight the need for targeted policies that extend beyond financial assistance to include food literacy education, affordable and nutritionally balanced campus meals, and culturally inclusive food environments. In particular, institutional support mechanisms such as scholarship programmes and opportunities for student employment may play an important role in mitigating economic barriers that influence dietary diversity and food access among international students.

Given that meal skipping was associated with both food insecurity and dietary variety, interventions should address structural determinants such as food affordability, time constraints, and access to culturally appropriate foods, rather than focusing solely on individual behaviours. Institutional food environments, including campus catering systems and retail access, warrant systematic evaluation to ensure equitable availability of nutritious options for diverse student populations. Universities may also benefit from integrating food security screening and referral mechanisms within student support services to identify students experiencing food-related hardship at an early stage. Psychosocial dimensions of food insecurity, including stigma and stress, should be integrated into student support services alongside mental health provision [51]. More broadly, national higher education policies should recognise food insecurity among international students as a structural public health issue that intersects with financial support systems, housing affordability, and student welfare frameworks.

## 6. Strengths and Limitations

This study has several strengths, including a robust sample size, the use of validated instruments to assess food insecurity and dietary diversity (FIES, FGDS, and FVS), and the application of complementary analytical approaches, including multivariable regression and principal component analysis, enabling a multidimensional assessment of dietary behaviour.

The cross-sectional design limits causal inference, and self-reported dietary measures may be subject to recall bias. Although food insecurity severity was categorised using established FIES thresholds, temporal variation and potential seasonal fluctuations in food access across the academic year were not examined. Additionally, academic performance indicators such as grade attainment or dropout intentions were not assessed, and mental health measures were not included in the present analysis. Scholarship and employment status may reflect both financial resources and underlying selection processes, and although statistical adjustments for relevant covariates were conducted, residual confounding cannot be entirely excluded.

While anthropometric and lifestyle-related variables were collected and are being examined in complementary analyses, the current manuscript focuses specifically on dietary diversity outcomes. Future longitudinal research integrating academic outcomes, mental health indicators, seasonal variation, and economic modelling of institutional interventions would provide a more comprehensive understanding of the structural and health consequences of food insecurity among international students. In addition, qualitative research exploring students’ lived experiences of food access and coping strategies may provide further insight into the contextual factors influencing dietary behaviours within international student populations.

## 7. Conclusions

This study provides evidence that food insecurity is prevalent and associated with constrained dietary patterns among international students in Hungary, even in contexts where the diversity of food groups appears adequate. By separating food group diversity from food variety and integrating behavioural, socioeconomic, and dietary pattern analyses, the findings reveal hidden nutritional vulnerabilities not fully captured through traditional indicators of food access.

While financial assistance, scholarship support, and part-time employment appear to mitigate some dietary constraints, behavioural adaptations and structural barriers continue to shape dietary adequacy. Socioeconomic resources such as income, employment, and institutional financial support were important predictors of dietary diversity in the adjusted analyses, indicating that economic capacity plays a central role in shaping dietary opportunities for international students.

These results emphasise the need for inclusive, equity-driven strategies that combine financial support with nutrition education, culturally sensitive food services, and structural interventions within university settings. Addressing food insecurity among international students is essential not only for improving dietary quality and well-being but also for supporting academic success and advancing broader public health goals of food and nutrition security.

Future research should adopt longitudinal and mixed-methods approaches to inform the design of sustainable, student-centred interventions in higher education. Such approaches may also help clarify the complex pathways linking food insecurity, socioeconomic conditions, and dietary behaviours among internationally mobile student populations.

## Figures and Tables

**Table 1:** Sociodemographic and socioeconomic characteristics of international students in Hungary (*n* = 380).

Variables	Category	*n*	%
Gender	Male	193	51
	Female	187	49
Age	18–25	207	55
	26–30	129	34
	>30	44	12
Race	African/African American	222	58
	Asian	100	26
	Spanish/Hispanic	8	2
	White/Caucasian	50	13
Regional distribution in Hungary	Central Hungary	137	36
	Northern Hungary	39	10
	Northern Great Plain	145	38
	Southern Transdanubia	28	7
	Southern Great Plain	31	8
Accommodation	Renting alone	100	26
	Shared housing (free)	20	5
	Shared housing (paid)	169	45
	University dormitory	91	24
Scholarship status	Not on scholarship	170	45
	Scholarship recipient	210	55
Level of study	Postgraduate	180	48
	Undergraduate	200	53
Employment status	Employed, full-time	17	5
	Self-employed	8	2
	Student, unemployed	269	71
	Working part-time	86	23
Monthly income (€)	≤€265	77	20
	€290–€400	92	24
	€425–€530	114	30
	€555–€795	72	19
	>€795	25	7

Percentages may not sum to 100% due to rounding or multiple response options.

**Table 2:** Food security and dietary diversity characteristics of the participants.

Variables	Category	*n*	%
Food security status	Food secure (FIES 0–3)	144	38
	Moderately food insecure (FIES 4–6)	117	31
	Severely food insecure (FIES 7–8)	119	31
Food group diversity	High Dietary Diversity (≥7 food groups)	367	97
	Medium Dietary Diversity (4–6 food groups)	13	3
Food variety categories	High Food Variety (68–101 items)	117	31
	Medium Food Variety (34–67 items)	190	50
	Low Food Variety (0–33 items)	73	19

**Table 5:** Association between food security status and sociodemographic and behavioural variables among international students in Hungary (*n* = 380).

Variable	χ^2^ (df, n)	*p*-Value	Cramer’s V
Gender	χ^2^(2, 380) = 5.40	0.067	0.12
Scholarship	χ^2^(2, 380) = 6.12	0.047	0.13
Employment	χ^2^(8, 380) = 7.05	0.531	0.10
Meal Skipping	χ^2^(2, 380) = 314.87	<0.001	0.91

χ^2^ = chi-square statistic; df = degrees of freedom; n = sample size.

**Table 6:** One-way ANOVA for FGDS and FVS across food security levels among international students in Hungary (*n* = 380).

Dependent Variable	Food Secure (n = 144)	Moderately Food Insecure (n = 117)	Severely Food Insecure (n = 119)	F(2,377)	*p*-Value
FGDS	11.2 ± 1.1	10.7 ± 1.1	10.9 ± 1.2	5.18	0.006
FVS	58.7 ± 22.9	55.0 ± 23.4	56.3 ± 23.7	0.89	0.411

Abbreviations: FGDS = Food Group Diversity Score; FVS = Food Variety Score. Data are presented as mean ± standard deviation (SD). Levene’s test indicated homogeneity of variances for both outcomes (*p* > 0.05).

**Table 7:** Factors associated with dietary diversity indicators (FGDS and FVS) among international students (*n* = 380).

Model 1: Adjusted for Sociodemographic, Behavioural and Economic Variables				
Predictor Variable	FGDS (β)	*p*-Value	FVS (β)	*p*-Value
Food security status	0.01	0.267	0.08	0.361
Monthly income	0.17	0.001	0.06	0.280
Monthly food expenditure	–0.05	0.342	−0.02	0.671
Age group	0.09	0.098	0.05	0.297
Gender	0.01	0.914	0.00	0.952
Scholarship status	0.14	0.006	0.28	<0.001
Employment status	0.10	0.063	0.20	<0.001
Meal-skipping	–0.26	0.004	−0.19	0.033

Model fit: FGDS: R^2^ = 0.11; Adjusted R^2^ = 0.09; F(8,371) = 5.82; *p* < 0.001. FVS: R^2^ = 0.13; Adjusted R^2^ = 0.11; F(8,371) = 7.10; *p* < 0.001. Dependent variables: FGDS and FVS. All predictors were entered simultaneously using the Enter method. Age was treated as a categorical variable (reference: youngest age group). Monthly income and monthly food expenditure were entered as continuous variables measured in euros (€), although grouped categories are presented descriptively in Table 1. Scholarship status was coded as scholarship-funded versus non-scholarship funded (reference). Employment status was coded with unemployed as the reference category.

## Data Availability

All data generated and analysed during the current study are not publicly available due to the need to protect participants’ privacy and confidentiality, but are available from the corresponding author upon reasonable request.

## Supplementary Materials

The following supporting information can be downloaded at: https://www.mdpi.com/article/10.3390/nu18060946/s1, Figure S1: Corrected item–total correlations for the eight Food Insecurity Experience Scale (FIES) items among international students in Hungary; Table S1: Internal consistency of the Food Insecurity Experience Scale (FIES) among international students; Table S2: Factor analysis results for the Food Insecurity Experience Scale (FIES) among international students; Table S3: Subgroup comparisons of FGDS and FVS according to student characteristics; Table S4: Multiple linear regression analysis for FGDS (*n* = 380); Table S5: Multiple linear regression analysis for FVS (*n* = 380); Figure S2A: Histogram of regression standardised residuals for FGDS; Figure S2B: Scatterplot of standardised residuals vs. predicted values for FGDS; Figure S3A: Histogram of standardised residuals for the FVS model; Figure S3B: Scatterplot of standardised residuals versus predicted values for the FVS model; Figure S4: Normal P–P plots showing the distribution of standardised residuals for the FGDS and FVS regression models.

## Abbreviations

The following abbreviations are used in this manuscript:

ANOVA	Analysis of Variance
DD	Dietary Diversity
DDS	Dietary Diversity Score
FAO	Food and Agriculture Organization
FFQ	Food Frequency Questionnaire
FGDS	Food Group Diversity Score
FIES	Food Insecurity Experience Scale
FSS	Food security status
FVS	Food Variety Score
IQR	Interquartile range
KMO	Kaiser–Meyer–Olkin
VIF	Variance Inflation Factor

## References

[1] Food Insecurity in Higher Education: A Contemporary Review of Impacts and Explorations of Solutions. https://www.mdpi.com/1660-4601/20/10/5884.

[2] Amoyaw J., Pandey M., Maina G., Li Y., Nkrumah D.O. (2022). Food Insecurity among Postsecondary International Students: A Scoping Review Protocol. BMJ Open.

[3] (2001). The State of Food Insecurity in the World. https://www.fao.org/4/y1500e/y1500e00.htm.

[4] Savoie-Roskos M.R., Hood L.B., Hagedorn-Hatfield R.L., Landry M.J., Patton-López M.M., Richards R., Vogelzang J.L., Qamar Z., OoNorasak K., Mann G. (2023). Creating a Culture That Supports Food Security and Health Equity at Higher Education Institutions. Public Health Nutr..

[5] Dewit J. (2020). Internationalization in Higher Education: Global Trends and Recommendations for Its Future. Policy Rev. High. Educ..

[6] International Students. https://www.migrationdataportal.org/themes/international-students-trends.

[7] Jin R., Le T.-T., Vuong T.-T., Nguyen T.-P., Hoang G., Nguyen M.-H., Vuong Q.-H. (2023). A Gender Study of Food Stress and Implications for International Students Acculturation. World.

[8] Ellison B., Bruening M., Hruschka D.J., Nikolaus C.J., van Woerden I., Rabbitt M.P., Nickols-Richardson S.M. (2021). Viewpoint: Food Insecurity among College Students: A Case for Consistent and Comparable Measurement. Food Policy.

[9] Coffino J.A., Spoor S.P., Drach R.D., Hormes J.M. (2021). Food Insecurity among Graduate Students: Prevalence and Association with Depression, Anxiety and Stress. Public Health Nutr..

[10] Marques B., Azevedo J., Rodrigues I., Rainho C., Gonçalves C. (2022). Food Insecurity Levels among University Students: A Cross-Sectional Study. Societies.

[11] Almoraie N.M., Alothmani N.M., Alomari W.D., Al-Amoudi A.H. (2025). Addressing Nutritional Issues and Eating Behaviours among University Students: A Narrative Review. Nutr. Res. Rev..

[12] Saaka M., Mutaru S., Osman S.M. (2021). Determinants of Dietary Diversity and Its Relationship with the Nutritional Status of Pregnant Women. J. Nutr. Sci..

[13] Verger E.O., Le Port A., Borderon A., Bourbon G., Moursi M., Savy M., Mariotti F., Martin-Prevel Y. (2021). Dietary Diversity Indicators and Their Associations with Dietary Adequacy and Health Outcomes: A Systematic Scoping Review. Adv. Nutr..

[14] Embling R., Pink A.E., Gatzemeier J., Price M., Lee M.D., Wilkinson L.L. (2021). Effect of Food Variety on Intake of a Meal: A Systematic Review and Meta-Analysis. Am. J. Clin. Nutr..

[15] Manikas I., Ali B.M., Sundarakani B. (2023). A Systematic Literature Review of Indicators Measuring Food Security. Agric. Food Secur..

[16] Lopes S.O., Abrantes L.C.S., Azevedo F.M., Morais N.D.S.D., Morais D.D.C., Gonçalves V.S.S., Fontes E.A.F., Franceschini S.D.C.C., Priore S.E. (2023). Food Insecurity and Micronutrient Deficiency in Adults: A Systematic Review and Meta-Analysis. Nutrients.

[17] Balan I.M., Gherman E.D., Brad I., Gherman R., Horablaga A., Trasca T.I. (2022). Metabolic Food Waste as Food Insecurity Factor—Causes and Preventions. Foods.

[18] Wright K.E., Lucero J.E., Ferguson J.K., Granner M.L., Devereux P.G., Pearson J.L., Crosbie E. (2021). The Impact That Cultural Food Security Has on Identity and Well-Being in the Second-Generation U.S. American Minority College Students. Food Sec..

[19] Odoms-Young A., Brown A.G.M., Agurs-Collins T., Glanz K. (2024). Food Insecurity, Neighborhood Food Environment, and Health Disparities: State of the Science, Research Gaps and Opportunities. Am. J. Clin. Nutr..

[20] Erturk S., Nguyen Luu L.A. (2022). Adaptation of Turkish International Students in Hungary and the United States: A Comparative Case Study. Int. J. Intercult. Relat..

[21] Ibiyemi T., Najam W., Oldewage-Theron W. (2024). Hungry, Stressed, and Away From “Home”: Predictors of Food Security and Perceived Stress Among International Students. Am. J. Health Promot..

[22] Császár Z., Tarrosy I., Dobos G., Varjas J., Alpek L. (2023). Changing Geopolitics of Higher Education: Economic Effects of Inbound International Student Mobility to Hungary. J. Geogr. High. Educ..

[23] Zemplényi L. (2022). Statistics on Education in Hungary. Hungarian Conservative.

[24] Hilal S., Kolozsvári L.R., Indrayathi P.A., Saeed S.N., Rurik I. (2024). Psychological Distress and Food Insecurity among International Students at a Hungarian University: A Post-Pandemic Survey. Nutrients.

[25] Chrzan-Rodak A., Bąk J., Chałdaś-Majdańska J., Machul M., Obuchowska A., Grzegorczyk A., Dziurka M., Ozdoba P., Dobrowolska B. (2024). Health-Related Behaviours of Foreign Students Studying in Poland and Their Determinants: A Mixed-Methods Study. Nutrients.

[26] Napier C. (2013). Evaluation of a Feeding Programme in Addressing Malnutrition in a Primary School.

[27] Swindale A., Bilinksy P. (2006). Household Dietary Diversity Score (HDDS) for Measurement of Household Food Access: Indicator Guide (Version 2).

[28] Oldewage-Theron W.H., Kruger R. (2008). Food Variety and Dietary Diversity as Indicators of the Dietary Adequacy and Health Status of an Elderly Population in Sharpeville, South Africa. J. Nutr. Elder..

[29] Hatløy A., Torheim L.E., Oshaug A. (1998). Food Variety—A Good Indicator of Nutritional Adequacy of the Diet? A Case Study from an Urban Area in Mali, West Africa. Eur. J. Clin. Nutr..

[30] Steyn N.P., Nel J.H., Nantel G., Kennedy G., Labadarios D. (2006). Food Variety and Dietary Diversity Scores in Children: Are They Good Indicators of Dietary Adequacy?. Public Health Nutr..

[31] Kennedy G., Ballard T., Dop M.C. (2011). Guidelines for Measuring Household and Individual Dietary Diversity.

[32] Food Insecurity Experience Scale|Voices of the Hungry|Food and Agriculture Organization of the United Nations. https://www.fao.org/in-action/voices-of-the-hungry/fies/en/.

[33] Sheikomar O.B., Dean W., Ghattas H., Sahyoun N.R. (2021). Validity of the Food Insecurity Experience Scale (FIES) for Use in League of Arab States (LAS) and Characteristics of Food Insecure Individuals by the Human Development Index (HDI). Curr. Dev. Nutr..

[34] El Zein A., Mathews A.E., House L., Shelnutt K.P. (2018). Why Are Hungry College Students Not Seeking Help? Predictors of and Barriers to Using an On-Campus Food Pantry. Nutrients.

[35] Nagata J.M., Palar K., Gooding H.C., Garber A.K., Whittle H.J., Bibbins-Domingo K., Weiser S.D. (2019). Food Insecurity Is Associated With Poorer Mental Health and Sleep Outcomes in Young Adults. J. Adolesc. Health.

[36] Soldavini J., Andrew H., Berner M. (2021). Characteristics Associated with Changes in Food Security Status among College Students during the COVID-19 Pandemic. Transl. Behav. Med..

[37] Sanci L., Williams I., Russell M., Chondros P., Duncan A.-M., Tarzia L., Peter D., Lim M.S.Y., Tomyn A., Minas H. (2022). Towards a Health Promoting University: Descriptive Findings on Health, Wellbeing and Academic Performance amongst University Students in Australia. BMC Public Health.

[38] Shi Y., Hayba N., Allman-Farinelli M. (2024). International Tertiary Education Students Experienced Difficulties in Dietary Transitions in Australia: A Qualitative Study. Health Promot. J. Aust..

[39] Shanab B.M., Khosla P., Hammad N.M., Steuart S.J., Brinker M., Sun G., Zadeh A.T., Gilbert D.T., Rooney B.L., Darko L. (2025). Food Insecurity Prevalence Among US Medical Students. JAMA Netw. Open.

[40] Wooten R., Spence M., Colby S., Steeves E.A. (2019). Assessing Food Insecurity Prevalence and Associated Factors among College Students Enrolled in a University in the Southeast USA. Public Health Nutr..

[41] Micevski D.A., Thornton L.E., Brockington S. (2014). Food Insecurity among University Students in Victoria: A Pilot Study. Nutr. Diet..

[42] Bruening M., Brennhofer S., van Woerden I., Todd M., Laska M. (2016). Factors Related to the High Rates of Food Insecurity among Diverse, Urban College Freshmen. J. Acad. Nutr. Diet..

[43] Webster J., Cornett A., Fletcher C. (2020). College Students and Mid-Month Eating Adjustments: Balancing Budgets Through Low Food Secure Behavior.

[44] Shi Y., Davies A., Allman-Farinelli M. (2021). The Association Between Food Insecurity and Dietary Outcomes in University Students: A Systematic Review. J. Acad. Nutr. Diet..

[45] Bolo A., Verger E., Fouillet H., Mariotti F. (2024). Exploring Multidimensional and Within-Food Group Diversity for Diet Quality and Long-Term Health in High-Income Countries. Adv. Nutr..

[46] Embling R., Price M.J., Lee M.D., Jones A., Wilkinson L.L. (2023). Associations between Dietary Variety, Portion Size and Body Weight: Prospective Evidence from UK Biobank Participants. Br. J. Nutr..

[47] Gómez M.I., Ricketts K.D. (2013). Food Value Chain Transformations in Developing Countries: Selected Hypotheses on Nutritional Implications. Food Policy.

[48] Merhout F., Doyle J. (2019). Socioeconomic Status and Diet Quality in College Students. J. Nutr. Educ. Behav..

[49] Vargas C., Backholer K., Bennett R., Maganti R., Lewis M., Marshall J., Sacks G., Alston L., Gupta A., Needham C. (2025). Working towards Affordable Healthy Diets: A Review on Innovations in Food Price Monitoring, Policy and Research in Australia and Beyond. Proc. Nutr. Soc..

[50] DeBate R., Himmelgreen D., Gupton J., Heuer J.N. (2021). Food Insecurity, Well-Being, and Academic Success among College Students: Implications for Post COVID-19 Pandemic Programming. Ecol. Food Nutr..

[51] Hattangadi N., Vogel E., Carroll L.J., Côté P. (2019). “Everybody I Know Is Always Hungry…But Nobody Asks Why”: University Students, Food Insecurity and Mental Health. Sustainability.

