# Supplementary material for: Adequate Dietary Diversity Versus Suboptimal Diet Quality: The Paradox of Food Insecurity Among International Students in Hungary
Source: Nutrients. 2026 Mar 17;18(6):946. doi: 10.3390/nu18060946 (PMC13029449; doi:10.3390/nu18060946)
Supplement: Supplementary file 1 [file nutrients-18-00946-s001.zip › nutrients-4137642-supplementary.pdf]

SUPPLEMENTARY MATERIAL

Table S1. Internal Consistency of the Food Insecurity Experience Scale among International Students (n = 380)

FIES Item	Corrected Item-Total Correlation	Cronbach's α if Item Deleted
Were you not able to eat the kinds of foods you prefer because of a lack of resources?	0.683	0.887
Did you eat a limited variety of foods due to lack of resources?	0.674	0.888
Did you eat some foods you did not want to eat because of a lack of resources to obtain others?	0.675	0.888
Did you have to eat a smaller meal than you felt you needed because there was not enough food?	0.753	0.880
Did you have to eat fewer meals per day because there was not enough food?	0.804	0.875
Was there ever no food to eat because of lack of resources to obtain more?	0.708	0.885
Did you go to sleep hungry at night because there was not enough food?	0.625	0.893
Did you go a whole day and night without eating anything because there was not enough food?	0.560	0.898

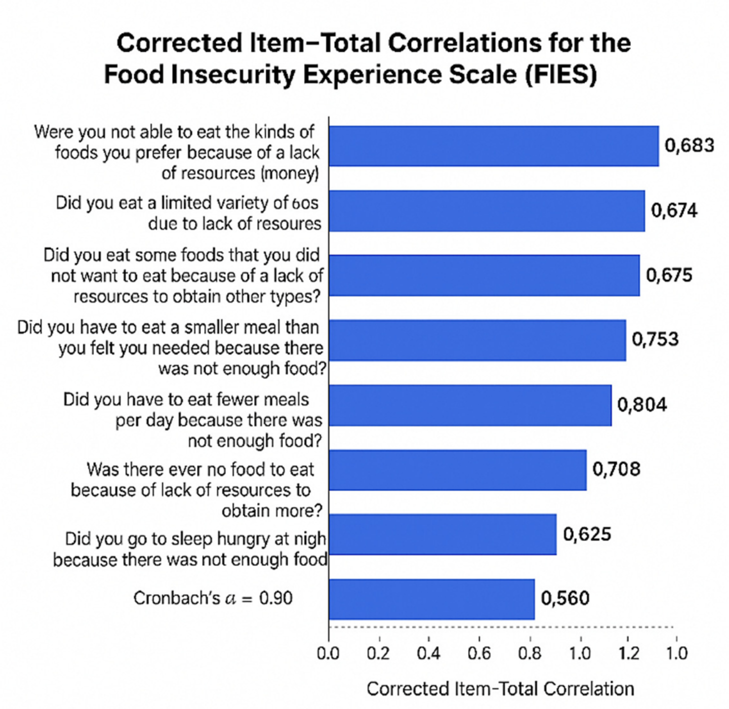

Figure S1. Corrected item–total correlations for the eight Food Insecurity Experience Scale (FIES) items among international students in Hungary (n = 380). All items exceeded the minimum acceptable correlation of 0.30, indicating strong internal consistency (Cronbach's $\alpha = 0.90$).

The eight-item Food Insecurity Experience Scale (FIES) demonstrated excellent internal reliability (Cronbach's $\alpha = 0.90$). Item-total correlations ranged from 0.56 to 0.80, and the removal of any item did not improve reliability ($\alpha = 0.88–0.90$). These results confirm that all items contributed meaningfully to the overall construct, supporting the reliability of the FIES for assessing food insecurity within a multicultural university population.

Table S2. Factor Analysis Results for the Food Insecurity Experience Scale (FIES) among International Students (n = 380)

Indicator	Statistic / Loading	Interpretation
Kaiser–Meyer–Olkin	0.859	Sampling adequacy = *meritorious*
Bartlett's Test of Sphericity	$\chi^2(28) = 2088.33$, $p < 0.001$	Items share common variance → suitable for factor analysis
Eigenvalue (Factor 1)	4.87	Explains 60.9 % of total variance
Communalities (range)	0.56 – 0.82	Acceptable to strong shared variance
Highest loading	0.83 ("Ate fewer meals per day")	Strong representation of the latent construct
Lowest loading	0.56 ("Went a whole day without eating")	Still acceptable (> 0.40)

Exploratory factor analysis confirmed the unidimensional structure of the FIES. Sampling adequacy was high (KMO = 0.86), and Bartlett's Test of Sphericity was significant ($\chi^2(28) = 2088.33$, $p < 0.001$). One factor emerged (eigenvalue = 4.87) accounting for 60.9 % of the total variance, with factor loadings ranging from 0.56 to 0.83. These results indicate that the FIES items coherently measured a single latent construct of food insecurity in this sample.

Summary of Test 1 (Reliability + Validity):

- Cronbach's $\alpha = 0.90$ → Excellent internal consistency
- KMO = 0.86, Bartlett p < 0.001 → Factorable data
- One factor explains ≈ 61 % variance → Strong construct validity

Table S3. Subgroup comparisons of FIES, FGDS, and FVS scores according to student characteristics.

Student characteristic	Subgroup	n	FGDS mean ± SD	p-value	FVS mean ± SD	p-value
**Gender**	Female	187	10.95 ± 1.09	0.976	56.43 ± 22.02	0.761
	Male	193	10.94 ± 1.22		57.16 ± 24.49	
**Age group**	18–25	207	10.84 ± 1.24	0.091	55.19 ± 23.72	0.251
	26–30	129	11.02 ± 1.08		57.91 ± 22.53	
	>30	44	11.23 ± 0.94		61.09 ± 23.14	
**Scholarship status**	Scholarship	210	10.84 ± 1.15	0.046	51.86 ± 20.85	<0.001
	Non-scholarship	170	11.08 ± 1.15		62.89 ± 24.70	
**Level of study**	Undergraduate	200	10.96 ± 1.21	0.787	59.91 ± 24.56	0.006
	Postgraduate	180	10.93 ± 1.10		53.34 ± 21.31	
**Continent of origin**	Africa	242	10.91 ± 1.25	0.145	56.36 ± 23.82	0.659
	Asia	104	11.10 ± 1.00		58.59 ± 22.83	
	Europe	27	10.78 ± 0.89		56.07 ± 21.64	
	The Americas	7	10.57 ± 0.98		48.14 ± 17.46	
**Accommodation**	Dormitory	91	10.76 ± 1.25	0.280	50.36 ± 22.43	0.012
	Shared	169	10.94 ± 1.08		57.08 ± 23.08	
	Renting	100	11.05 ± 1.13		60.30 ± 22.62	
	Living free	17	11.29 ± 1.49		67.12 ± 28.09	
	Home	3	11.33 ± 1.16		60.67 ± 16.26	
**Monthly income group**	≤ €265	77	10.25 ± 1.19	<0.001	39.04 ± 15.25	<0.001
	€290–€400	92	11.10 ± 1.09		67.76 ± 26.79	
	€425–€530	114	11.15 ± 1.18		59.81 ± 21.18	
	€555–€795	72	11.03 ± 0.99		55.83 ± 20.42	
	>€795	25	11.36 ± 0.81		60.20 ± 14.97	
**Hungarian region**	Central Hungary	137	11.05 ± 1.11	0.008	59.56 ± 22.84	<0.001
	Northern Hungary	38	10.58 ± 1.18		47.87 ± 22.86	

Student characteristic	Subgroup	n	FGDS mean ± SD	p-value	FVS mean ± SD	p-value
	Northern Great Plain	145	11.01 ± 1.13		55.21 ± 22.08	
	Southern Great Plain	31	11.16 ± 1.07		73.94 ± 21.58	
	Southern Transdanubia	28	10.36 ± 1.39		45.07 ± 22.30	
**Employment status**	Unemployed	269	10.86 ± 1.19	0.014	54.26 ± 22.39	0.001
	Part-time	86	11.02 ± 1.10		60.22 ± 24.51	
	Full-time	17	11.47 ± 0.94		72.71 ± 22.46	
	Self-employed	8	11.88 ± 0.35		71.50 ± 21.18	

Results for subgroups with small sample sizes (e.g., n < 10) should be interpreted cautiously.

Table S4. Multiple linear regression analysis for FGDS (n = 380)

**Model: Adjusted for sociodemographic, behavioural and economic variables**							
**Predictor Variable**	**B**	**SE B**	**β**	**t**	**p-value**	**95% CI for B**	**VIF**
**Constant**	9.22	0.56	—	16.57	<0.001	8.13 to 10.32	—
**Food security status**	0.14	0.12	0.01	1.11	0.267	−0.11 to 0.38	3.28
**Estimated monthly income**	0.000002	0.000001	0.17	3.21	**0.001**	0.000001 to 0.000004	1.22
**Monthly food expenditure**	−0.000003	0.000003	−0.05	−0.95	0.342	−0.000009 to 0.000003	1.13
**Age**	0.03	0.02	0.09	1.66	0.098	−0.01 to 0.06	1.15
**Gender**	0.01	0.12	0.01	0.11	0.914	−0.21 to 0.24	1.03
**Scholarship status**	0.33	0.12	0.14	2.76	**0.006**	0.09 to 0.56	1.08
**Employment status**	0.18	0.09	0.10	1.86	0.063	−0.01 to 0.36	1.24
**Meal skipping**	−0.61	0.21	−0.26	−2.88	**0.004**	−1.02 to −0.19	3.30

Model fit: $R^2 = 0.11$; Adjusted $R^2 = 0.09$; $F(8,371) = 5.82$; $p < 0.001$

Independent variables were entered simultaneously (Enter method). Bold p-values indicate statistical significance ($p < 0.05$. Tolerance > 0.80and VIF <5 indicate no evidence of problematic multicollinearity.

Table S5. Multiple linear regression analysis for FVS (n = 380)

**Model: Adjusted for sociodemographic, behavioural and economic variables**							
**Predictor Variable**	**B**	**SE B**	**β**	**t**	**p-value**	**95% CI for B**	**VIF**
Constant	17.10	11.06	—	1.55	0.123	−4.65 to 38.85	—
Food security status	2.25	2.46	0.08	0.92	0.361	−2.58 to 7.08	3.28
Estimated monthly income	0.000015	0.000014	0.06	1.08	0.280	−0.000012 to 0.000043	1.22
Monthly food expenditure	−0.000027	0.000064	−0.02	−0.43	0.671	−0.000153 to 0.000099	1.13
Age	0.37	0.35	0.05	1.04	0.297	−0.32 to 1.05	1.15
Gender	0.14	2.28	0.00	0.06	0.952	−4.35 to 4.62	1.03
Scholarship status	13.23	2.35	0.28	5.62	<0.001	8.60 to 17.86	1.08
Employment status	7.06	1.87	0.20	3.78	<0.001	3.39 to 10.73	1.24
Meal skipping	−8.96	4.18	−0.19	−2.15	0.033	−17.18 to −0.75	3.30

Model fit: $R^2 = 0.13$; Adjusted $R^2 = 0.11$; $F(8, 371) = 7.10$, $p < 0.001$

Note: Independent variables were entered simultaneously using the Enter method. **Bold p-values indicate statistical significance (p < 0.05). Variance inflation factor (VIF) values < 5 indicate no evidence of problematic multicollinearity.**

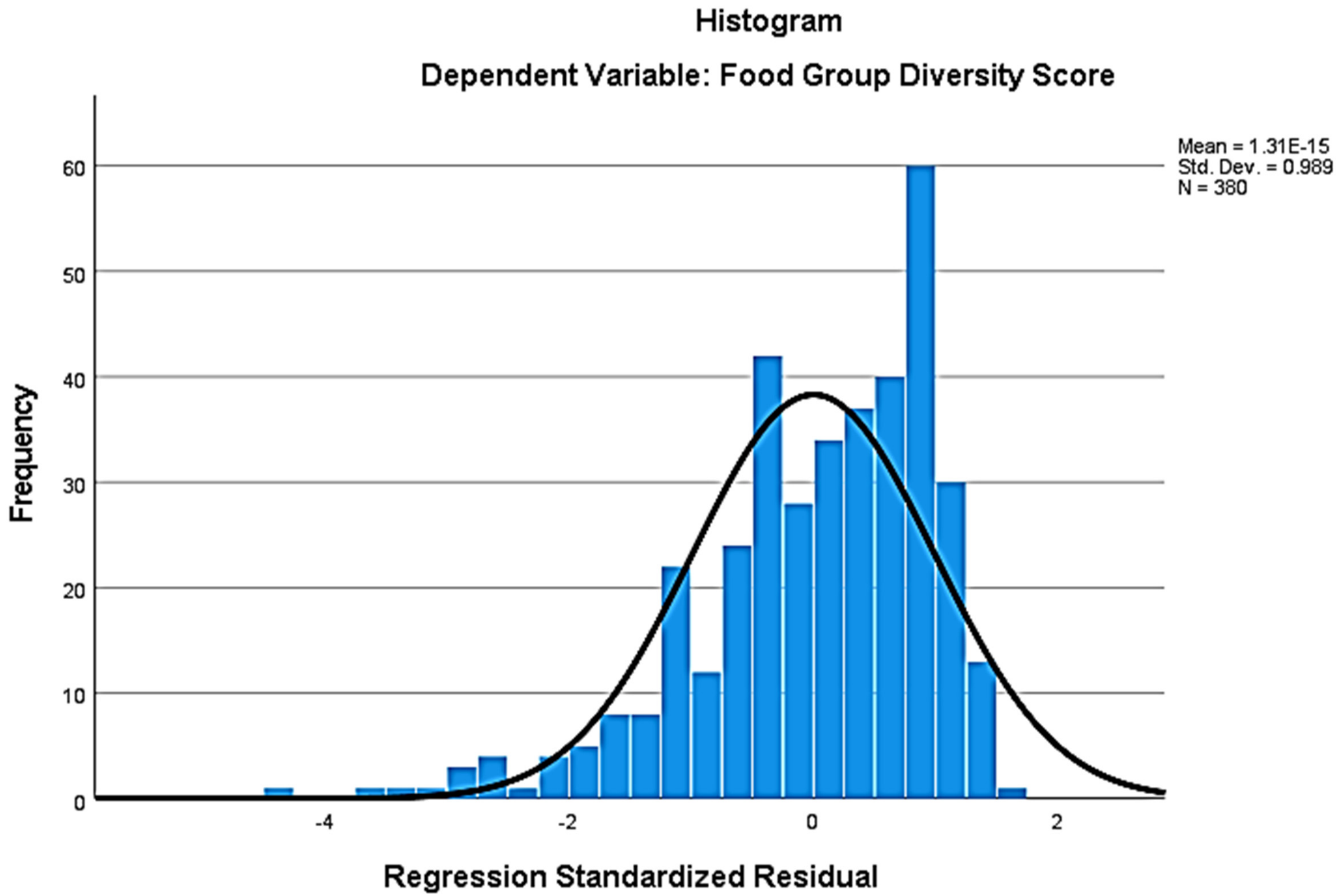

Figure S2A. Histogram of Regression Standardised Residuals for FGDS

Histogram of regression standardised residuals for the FGDS model (n = 380).

Residuals are approximately normally distributed around zero (Mean = -1.38×10^{-15}, SD = 0.991), confirming the assumption of normality for model residuals.

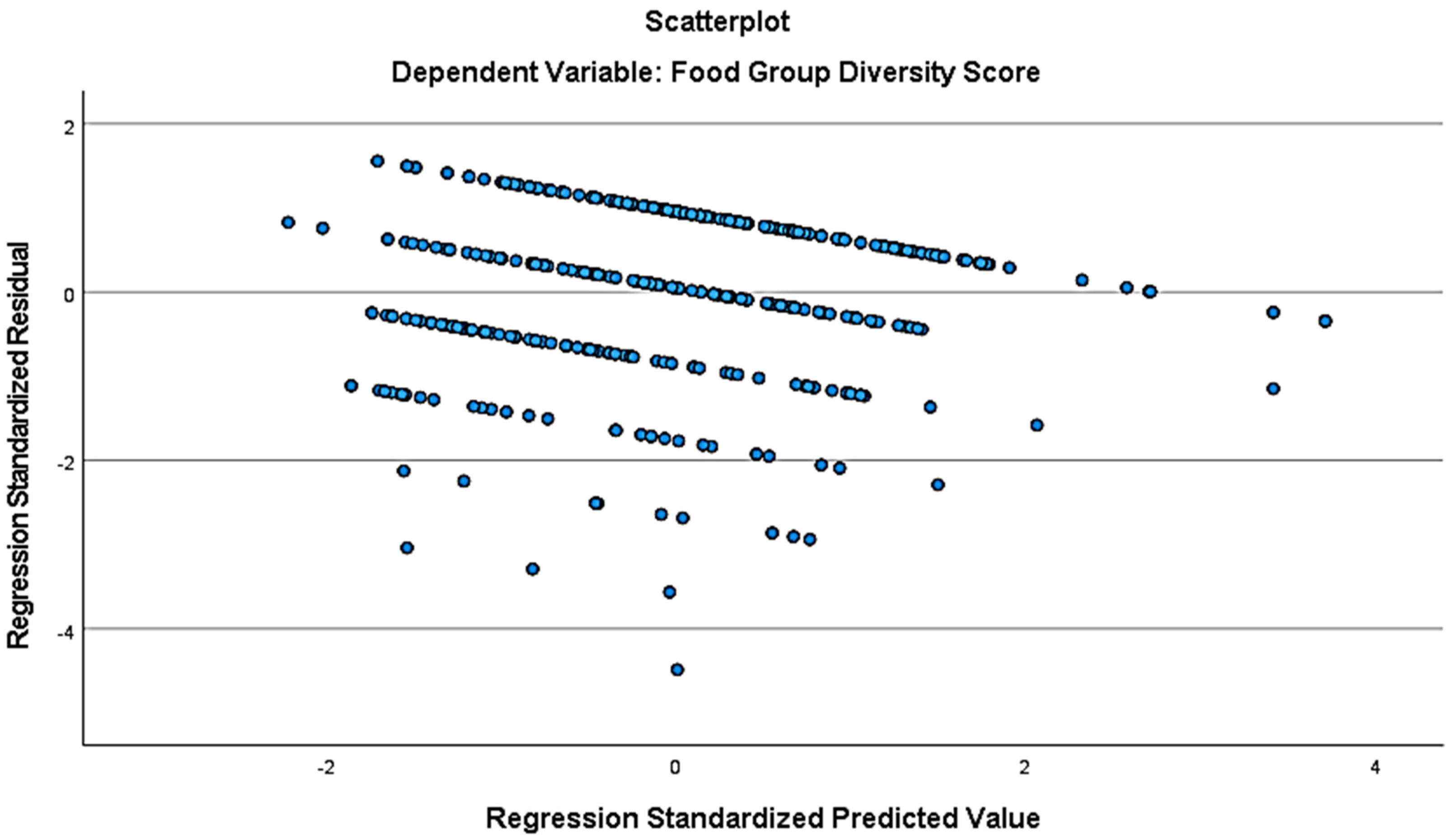

Figure S2B. Scatterplot of Standardised Residuals vs Predicted Values for FGDS

Scatterplot of standardised residuals against standardised predicted values for the FGDS model.

The random dispersion of points around the zero line suggests that assumptions of linearity and homoscedasticity were met, with no visible pattern or funnel shape.

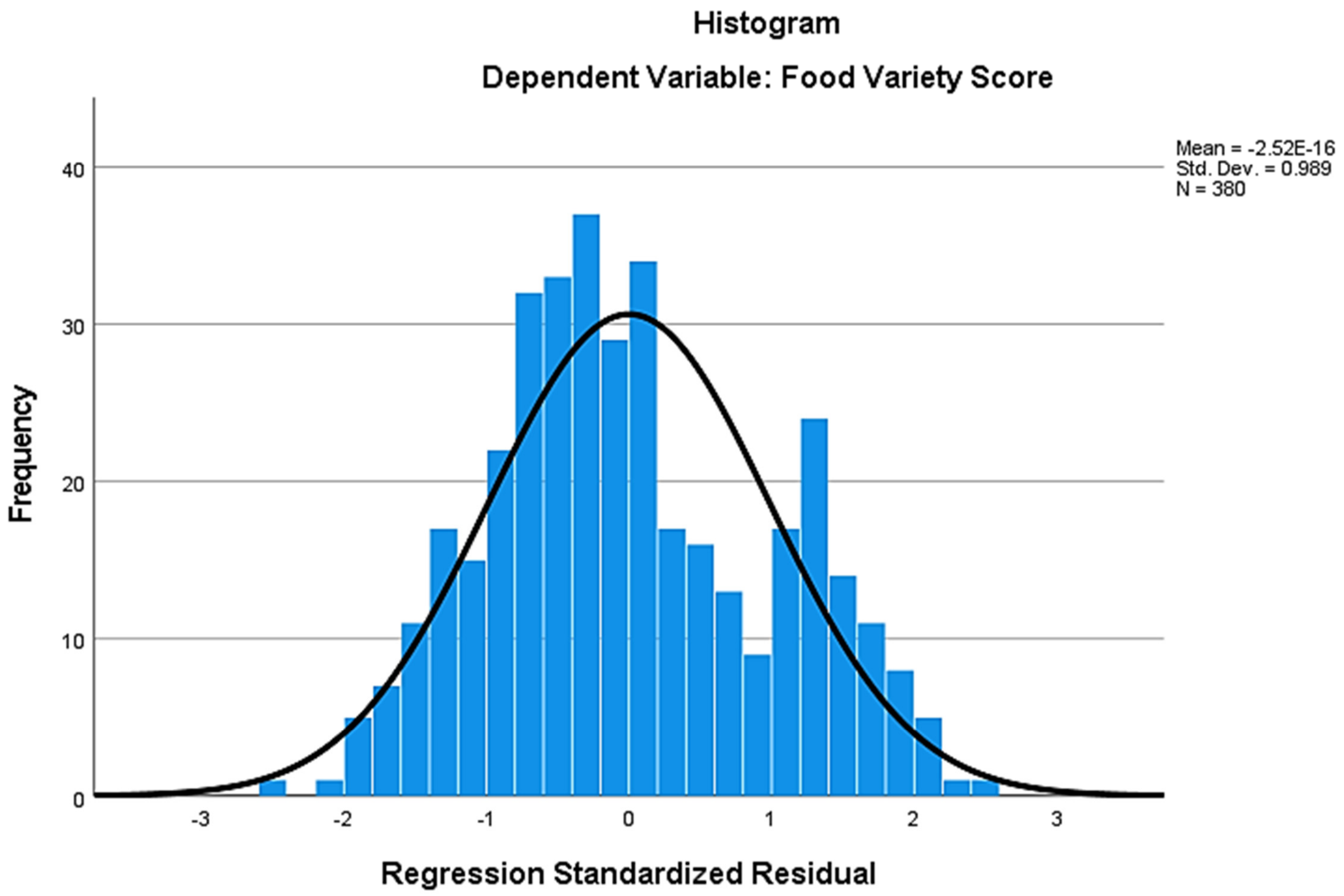

Figure S3A. Histogram of Standardised Residuals for the FVS Model.
Residuals are approximately normally distributed with a mean near zero and a standard deviation close to 1, supporting the assumption of normality.

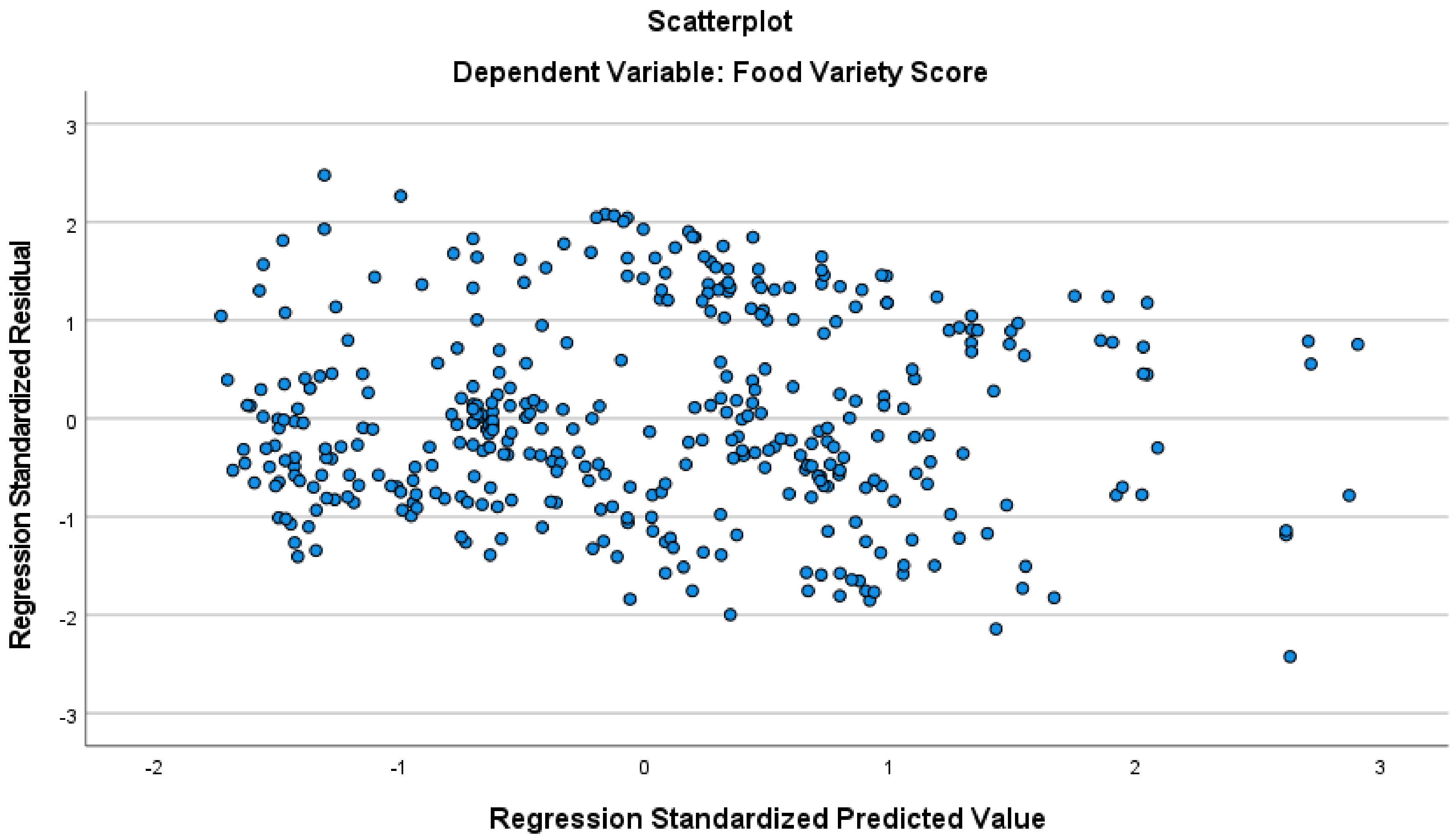

Figure S3B. Scatterplot of Standardised Residuals versus Predicted Values for the FVS Model.

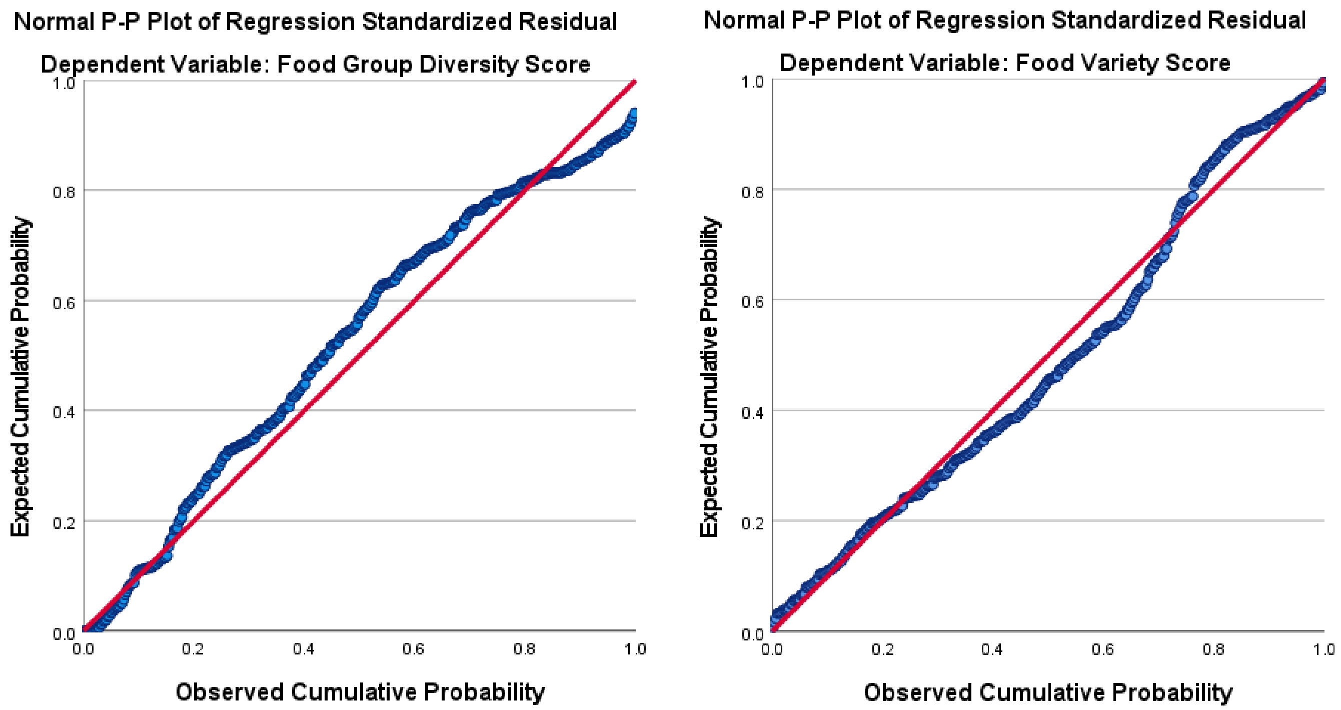

Figure S4. Normal P–P plot showing the distribution of standardised residuals for the FGDS regression model and Normal P–P plot showing the distribution of standardised residuals for the FVS regression model.
